# Evaluating ChatGPT for converting clinic letters into patient-friendly language: a quantitative study

**DOI:** 10.3399/BJGPO.2024.0300

**Published:** 2025-07-16

**Authors:** Simon C Cork, Keith Hopcroft

**Affiliations:** 1 School of Medicine, Anglia Ruskin University, Bishop Hall Road, Chelmsford, UK; 2 Laindon Medical Group, Basildon, UK

**Keywords:** Family practice, Hospital referrals, Patient perspectives, Primary healthcare, General practitioners, Artificial Intelligence, Generative AI

## Abstract

**Background:**

Previous research has shown that communication with patients in language they understand leads to greater comprehension of treatment and diagnoses, but can be time consuming for clinicians.

**Aim:**

We sought to investigate the utility of ChatGPT-4 Classic in translating clinic letters into language patients understood without loss of clinical information, and to assess what impact this had on patients’ understanding of letter content.

**Design & setting:**

Single-blinded quantitative study using objective and subjective analysis of language complexity.

**Method:**

Twenty-three clinic letters were provided by consultants across eight specialties. Letters were inputted into ChatGPT-4 Classic with a prompt related to improve understanding for patients. Patient representatives were then asked to rate their understanding of the content of letters.

**Results:**

Translation of letters by ChatGPT-4 Classic resulted in no loss of clinical information, but did result in significant increase in understanding, satisfaction, and decrease in the need to obtain medical help to translate the letter contents by patient representatives compared with clinician-written originals.

**Conclusion:**

Overall, we concluded that ChatGPT-4 Classic can be used to translate clinic letters into patient-friendly language without loss of clinical content and that these letters are preferred by patients.

## How this fits in

ChatGPT has previously been shown to produce clinic letters from scratch in a small number of niche specialties, but such use limits their holistic nature. Moreover, this approach limits clinic letters to patient-friendly language only, which has been shown to be disadvantageous for primary care physicians. The impact on patients has also not been demonstrated. In this article, we demonstrated the ability of ChatGPT-4 Classic to translate clinic letters written in complex medical language into patient-friendly language from a range of specialities and evaluated its impact on patients understanding of the content of letters.

## Introduction

The importance of clear written communication between health professionals and patients has been high on the agenda politically and clinically for the last 24 years. The NHS Plan of 2000 recommended that, *‘patients should as of right receive copies of all correspondence between health professionals about their care’*.^1^ Additionally, in 2018, the Academy of Medical Royal Colleges produced guidance on writing outpatient clinic letters to patients that pointed out that good medical practice and the NHS Constitution stress the need to provide information to patients in a way they can understand.^2^ This guidance emphasised the benefit of writing directly to the patient, rather than sending them a copy of the GP letter. Indeed, the idea that ‘*all outpatient letters and discharge summaries that are currently written to GPs and copied to patients should be revised and written directly to patients with a copy to the GP*’ has been taken on as a quality improvement project by the Academy of Medical Royal Colleges.^1^

In 2022–23, there were 124.5 million outpatient appointments in England,^3^ each typically generating a clinic letter. The workload involved in creating patient-friendly letters, or patient-friendly versions of the GP letter, is potentially onerous, and perhaps explains why, in many cases, secondary care doctors still tend to simply copy patients into the letters they send to GPs. These are typically written in technical language with an inevitable risk of being, at best, unclear and confusing and, at worst, impenetrable and worrying. The result can be patient confusion and alarm, with a potential exacerbation of health inequalities, and an increase in GP workload via appointments arranged by patients to have these letters interpreted.^4^

The advent of generative AI, in forms such as ChatGPT, offers a possible solution. Research has shown that ChatGPT has potential in generating clinical letters, radiology reports, medical notes, and discharge summaries,^5^ albeit with some caveats about the quality of the material generated^6^ and the degree to which it can simplify patient information,^7^ and a warning that humans need to ‘stay in the loop’.^8,9^

In this study, we wished to explore whether clinic letters generated in secondary care could easily be converted by ChatGPT-4 Classic into a patient-friendly, personalised version without losing key clinical information, and whether this version is clearer both in terms of objective readability tests and end-user (the patient) assessment.

## Method

Consultant specialists were asked to provide fictional clinic letters in their normal writing style. A total of 23 letters were received (six ear, nose, and throat; six paediatric; four gastroenterology; two neurology; two psychiatry; one renal; one gynaecology; and one respiratory letter) for analysis. Most clinic letters received were addressed to doctors (*n* = 21/23, 91%), with two letters addressed directly to patients (*n* = 2/23, 9%), one for renal and respiratory specialities.

Each original letter was copied in its entirety and ChatGPT-4 Classic was given the following command:

*‘Convert the following letter into more easily understood language aimed at a UK-based patient with an average reading ability. The letter should be addressed to the patient and should maintain the tone of a strictly formal letter without any colloquialisms or conversational tone*.’

Letters related to paediatric patients were given the following modified command:

*‘Convert the following letter into more easily understood language aimed at a UK-based patient with an average reading ability. The letter should be addressed to the patient’s parent/guardian and should maintain the tone of a strictly formal letter without any colloquialisms or conversational tone*.’

For uniformity across all letters, the addressee and signatory were the same. For original letters, the addressee was changed to ’Dr F’ (in other words, *‘Dear Dr F*’) and the signatory was changed to ’Dr Y’, except for in the letters originally addressed to patients, in which the addressee was changed to ’Mr X’ or ’Ms X’. In ChatGPT-4 Classic outputs, the addressee was changed to either ’Mr X’ or ’Ms X’ (in other words, *‘Dear Mr X*’) and the signatory changed to ’Dr Y’.

Outputs from ChatGPT-4 Classic were analysed manually by a clinician for loss of clinical information relative to the original letter by circling clinical information in both letters. Original letters and outputs from ChatGPT-4 Classic were then subjected to both objective and subjective readability analysis.

Objective analysis was undertaken by comparing the Flesch-Kincaid, SMOG, Gunning Fog, Coleman-Liau, and Automated readability scores of the original letter and ChatGPT-4 Classic using freely available online calculators.

Subjective analysis was conducted using patient representatives. Patient representatives were recruited via email from Anglia Ruskin University School of Medicine Patient Partners database and were asked to participate in a questionnaire-based study investigating the clarity of clinic letters.

Online survey software (Online Surveys v2, JISC) was used to produce two separate surveys. Each survey contained an equal mixture of original letters and AI-generated outputs. The original letter and its corresponding AI-generated output were placed in separate surveys. A total of 15 patient representatives were recruited to each read through every letter in the survey they received and answer a series of questions related to their understanding of the content and how happy they would be to receive that letter as a patient, using a 5-point Likert scale (1 = strongly disagree, 5 = strongly agree). Patient representatives were recruited on the basis that they had no self-reported prior medical knowledge (no healthcare-related qualification). Each survey was sent to half of the patient representatives, such that no individual saw an original letter and its corresponding ChatGPT-4 Classic output.

Objective and subjective scores were analysed for statistical significance using paired *t*-tests. A *P* value of <0.05 was considered statistically significant.

## Results

The command used to convert standard clinic letters into patient-friendly versions resulted in no loss of clinical information nor introduction of any hallucinations (example letter shown in supplementary Figure 1).

The average length of AI-generated letters was significantly longer than original letters (average word length original: 244 ±23, AI: 348 ±13).

**Figure 1.**
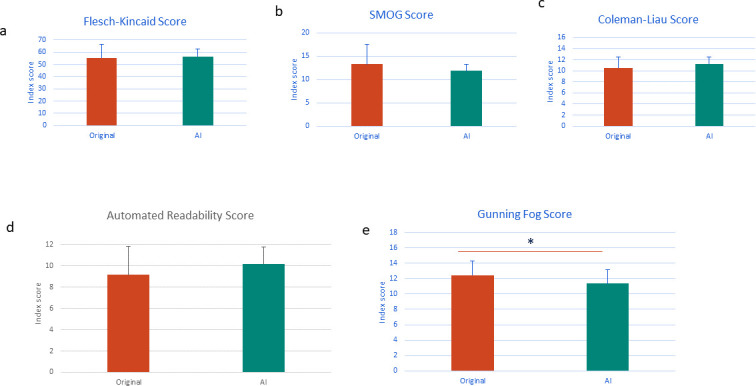
Objective readability scores Comparison of readability between original clinic letters and AI-generated outputs revealed no significant difference in **A:** Flesch-Kincaid score, **B:** SMOG score, **C:** Coleman-Liau score or **D:** Automatic readability score. A significant decrease in readability score of AI generated output (equivalent to a decrease in reading age) was observed in **E:** Gunning Fog score (*P*<0.05) compared with the original clinic letters. * *P*<0.05.

Objective analysis revealed no significant change in readability scores using the Flesch-Kincaid (*P* = 0·7), SMOG (*P* = 0·15), Coleman-Liau (*P* = 0·14), or automated readability models (*P* = 0·17) (Figure 1a–d). A significant decrease in readability score was observed in the Gunning Fog index (original: 12·44, AI: 11·35, *P* = 0·02), representing a drop from ’high school senior’ reading level to ’high school junior’ level (equivalent to a reading age of 17 and 16 years, respectively) (Figure 1e).

The demographic data for patient representatives involved in the subjective analysis are detailed in Table 1.

**Table 1.** Demographics of patient representatives

Demographic criteria	Patient representatives
Gender: female (*n*, %)	7, 47%
Age range, years (*n*)	35–44 = 1 45–54 = 1 55–64 = 4 ≥65 = 9
Education (*n*)	Secondary education = 3 Further education = 4 Degree or higher = 8
Ethnicity (*n*)	White/Caucasian = 13 Mixed/multiple ethnic groups = 2
English as first language: Yes (*n*, %)	15, 100%

**Figure 2.**
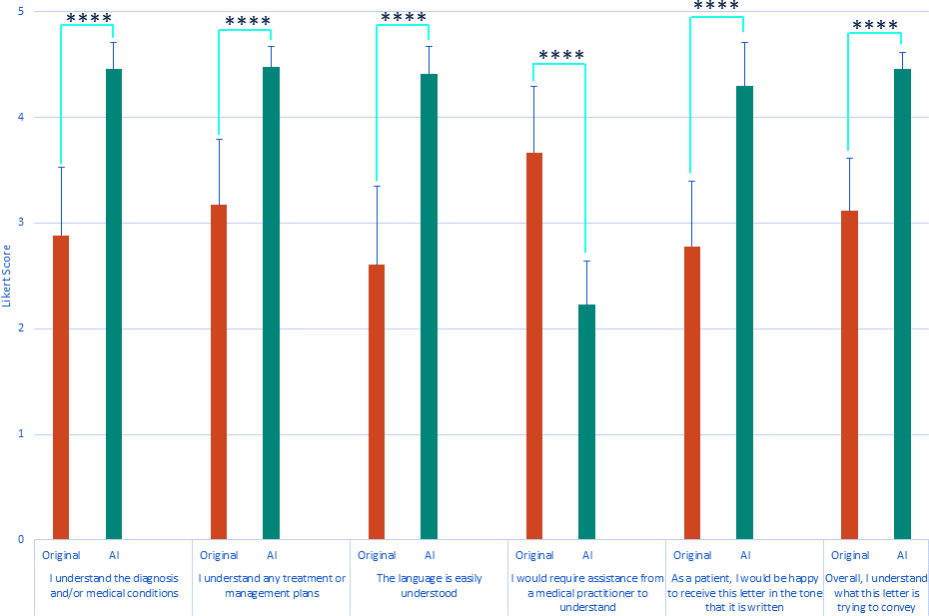
Results from patient representative analysis Patient representatives were presented with a mixture of original clinic letters and AI-generated outputs (but not the original and AI output of the same letter) and asked to rate their understanding of the content across a series of questions. There was a significant increase in understanding of content and approval of AI-generated outputs compared with original clinic letters. There was a significant decrease in the requirement for patient representatives to require medical assistance with understanding the content of AI-generated outputs compared with original clinic letters. ****P<0.0001

AI-generated letters led to significantly increased patient scores for understanding of diagnosis and/or medical conditions (original: 2·88 ±0·64, AI : 4·46 ±0·25, *P*<0·0001), understanding of treatment or management plans (original: 3·17 ±0·62, AI: 4·47 ±0·2, *P*<0·0001), understanding of language (original: 2·61 ±0·74, AI: 4·41 ±0·26, *P*<0·0001), satisfaction in receiving such a letter in the tone written (original: 2·78 ±0·62, AI: 4·30 ±0·41, *P*<0·0001), and overall understanding (original: 3·12 ±0·5, AI: 4·46 ±0·16, *P*<0·0001). There was a significant score reduction related to the requirement for assistance from a medical practitioner to understand the content of the letter in AI-generated letters compared to original letters (original: 3·67 ±0·63, AI: 2·23 ±0·42, *P*<0·0001, Figure 2).

Of note, two original letters were written addressed to the patient. Subjective analysis reveals a significant increase in the overall understanding of AI-generated interpretations of these letters compared to the original (original: 2.79 ±0.23, AI: 4.38 ±0.11, *P*<0.0001).

## Discussion

### Summary

This study demonstrated the capacity of generative AI, in this case ChatGPT-4 Classic, to take clinic letters addressed to clinicians and translate them into language that patients find easier to understand and more favourable, without any loss of clinical information.

The fact that only one objective readability index demonstrated a significant reduction in complexity was surprising but was likely due to the nature of how they attribute scores to language. These indexes consider sentence length and/or complexity. Given the nature of the explanations needed to generate patient-friendly versions, AI-generated letters were on average longer in length than originals, resulting in longer sentences. Of note, the index that demonstrated a significant decrease in complexity considered the number of words that use three or more syllables (defined as ’complex words’), demonstrating that although sentence length may have increased, the complexity of the language in AI-generated letters was reduced. This result highlighted the need to employ human end-user analysis when investigating whether AI-generated letters are considered easier to understand, rather than relying on objective readability indexes.

### Strengths and limitations

As far as we are aware, this study was the first of its kind to employ end-user analysis of letter contents to assess ease of understanding, rather than relying solely on objective readability indexes. This introduced significant strengths to the results; as demonstrated above, objective readability indexes do not necessarily match real patient views.

Our study was not without limitations, as the patient representatives were typically older (60% over 65 years of age), White (86%), and educated to degree level or higher (53%), which may not be representative of the wider population.

Moreover, while care was taken to ensure patient representatives were blinded as to the origin of each letter (original or AI-generated), it is possible, given that all AI generated letters were addressed specifically to patients, that a degree of unblinding occurred. This was mitigated against by ensuring that no participant received both the original and AI generated version of a particular letter and that some original letters were addressed to patients, rather than fellow clinicians. Nevertheless, while unlikely, some biasing of responses towards perceived AI-generated letters was possible.

Further work will be needed in a real-world setting to determine whether the results in this study, suggesting improved understanding by patients of clinical letters, will translate into actual improvements, such as reduced consulting for explanations of their contents.

Additionally, while our command prompt produced satisfactory results for our study, iterations on this command are likely to provide different responses, which would require further analysis. Further work might also explore the workload implications around the administration and human checking of such a system.

### Comparison with existing literature

Previous studies have also successfully used this technology, demonstrating in their case that production of patient-focused clinic letters using shorthand instructions without a corresponding original letter was possible, resulting in letters with high overall correctness and humanness scores written at a reading level similar to current real-world human written letters.^9^ One study showed that ChatGPT can simplify discharge letters, although the rating for patient-friendliness was derived using an unvalidated tool (the Patient Education Material Assessment Tool) as a proxy marker, and the process resulted in significant omissions and inaccuracies.^6^

However, AI conversion of actual clinic letters into patient-friendly versions has not previously been studied, nor has real end-user analysis been determined. In our study, only one objective readability index showed a significant decrease in score (equating to more easily understood language). Despite this, assessment by real end-users demonstrated a significant increase in understanding and a decrease in the need to consult medical professionals to translate the content of the letters. Our study was also the first to our knowledge to test the utility of generative AI to translate patient-focused clinic letters across several different specialties and to include end-user analysis.

Previous studies have demonstrated that simplification of patient correspondence leads to improved understanding of treatment and diagnoses.^10,11^ However, GPs prefer to receive letters written for them, citing a lack of terminology and relevant detail if they only receive a copy of the letter written to the patient.^11^ The results of this study demonstrated the utility of generative AI to take a letter containing technical language and transform it in a personalised way into patient-centred language, without generating additional workload for clinicians and without losing key clinical information. This means that both versions could be sent from the outpatient clinic, one to the patient and one to the GP, each version pitched correctly to the appropriate recipient, incurring minimal cost and effort. The result is likely to be improved communication with patients, enhanced patient satisfaction, and fewer primary care appointments wasted purely to interpret impenetrable or confusing letters from secondary care.

One limitation of generative AI is the risk of so-called ’hallucinations’, where information is provided based on inaccurate, misinterpreted, or fictitious information. Such hallucinations have the potential to cause at best alarm and at worst harm to patients if used in a healthcare setting. Hallucinations are more likely to be generated when the inputted information is limited. For example, a study investigating the use of AI to provide guidance on colonoscopy intervals in response to hypothetical patient scenarios, produced significantly fewer hallucinations when provided with specific information on which guidelines to follow.^12^ In our study, manual analysis of the generated letters revealed neither loss of clinical information nor hallucinations. Previous studies demonstrating the use of generative AI to produce patient-friendly clinical reports using similar methodology to this study have found this to be an issue.^13^ It is not clear why this was absent from the current study, but the version of ChatGPT or the prompt used are possible explanations. Nevertheless, the risk of hallucinations emphasises the importance of manually checking any generated clinic letters for accuracy.

### Implications for practice

Given the widespread availability of generative AI models such as ChatGPT, the implications of this study could be rapidly employed within healthcare organisations. However, care should be taken to ensure that generation of patient-focused clinic letters using ChatGPT (or other generative AI systems) complies with local personal data protection laws. Anonymising clinic letters prior to inputting them into ChatGPT may overcome these issues, but local guidance should be sought.

## Data

The data that support the findings of this study are available from the corresponding author on reasonable request.
